# Regulatory spine RS3 residue of protein kinases: a lipophilic bystander or a decisive element in the small-molecule kinase inhibitor binding?

**DOI:** 10.1042/BST20210837

**Published:** 2022-02-28

**Authors:** Ekaterina Shevchenko, Tatu Pantsar

**Affiliations:** 1 Department of Internal Medicine VIII, University Hospital Tübingen, Otfried-Müller-Strasse 14, Tübingen, DE 72076, Germany; 2 School of Pharmacy, Faculty of Health Sciences, University of Eastern Finland, Yliopistonranta 1, 70210 Kuopio, Finland

**Keywords:** kinase inhibitors, point mutations, protein kinases, protein–ligand interaction, regulatory-spine

## Abstract

In recent years, protein kinases have been one of the most pursued drug targets. These determined efforts have resulted in ever increasing numbers of small-molecule kinase inhibitors reaching to the market, offering novel treatment options for patients with distinct diseases. One essential component related to the activation and normal functionality of a protein kinase is the regulatory spine (R-spine). The R-spine is formed of four conserved residues named as RS1–RS4. One of these residues, RS3, located in the C-terminal part of αC-helix, is usually accessible for the inhibitors from the ATP-binding cavity as its side chain is lining the hydrophobic back pocket in many protein kinases. Although the role of RS3 has been well acknowledged in protein kinase function, this residue has not been actively considered in inhibitor design, even though many small-molecule kinase inhibitors display interactions to this residue. In this minireview, we will cover the current knowledge of RS3, its relationship with the gatekeeper, and the role of RS3 in kinase inhibitor interactions. Finally, we comment on the future perspectives how this residue could be utilized in the kinase inhibitor design.

## Introduction

**Figure 1.**
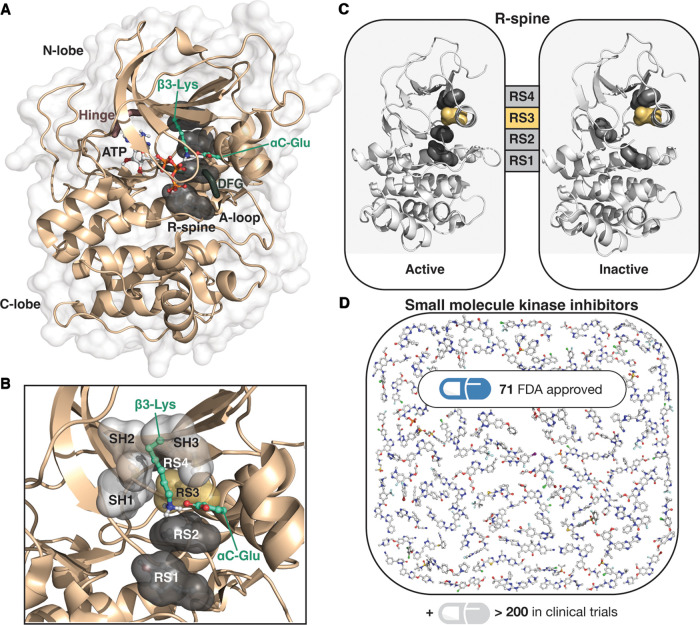
Protein kinase domains are important drug targets. (**A**) A typical structure of a protein kinase domain. ATP-binding cleft is located between the N- and C-lobes of the kinase. In the figure, structure of cAMP-dependent protein kinase catalytic subunit alpha is depicted (PDB ID: 4wb5 [20]; inhibitory peptide is hidden). R-spine residues are illustrated with black surface. ATP, β3-Lys and αC-Glu are shown with stick model. (**B**) Shell residues SH1–SH3 (grey surface) are located next to the R-spine RS3 and RS4 residues. Shell residue SH2 (gatekeeper) is located close to RS3 (yellow surface). (**C**) The R-spine of a protein kinase is assembled in active conformation and disassembled in inactive conformation. In the figure, active and inactive configurations are illustrated with BRAF (PDB IDs: 4e26 [21] and 1uwh [22]). (**D**) Several small-molecule kinase inhibitors are already in clinical use, and dozens are in clinical trials.

Protein kinases are dynamic proteins that regulate a multitude of cellular signalling processes. They control the activity of their downstream targets mainly by phosphorylation, and their own activity is also usually controlled in the same manner. The human kinome comprises more than 500 protein kinases [1], and nearly 500 proteins contain a typical kinase domain [2]. Still, the biological role of many protein kinases is largely unknown, and there are ongoing efforts aiming to characterize these poorly understood protein kinases [3]. Although protein kinases display high similarity in their kinase domain, there is a higher level diversity in their structures; while some kinases consist (almost) solely of the kinase domain (e.g. MAPK14, GSK3B), other are larger multidomain proteins (e.g. LRRK2 [4]). The structure and function of the protein kinase domain is well-established (Figure 1A). For a comprehensive view of structural history of protein kinases, the reader is recommend a recent review by Taylor et al. [5].

In the protein kinase domain, one of the key dynamic elements in regulating protein kinase function is the hydrophobic regulatory-spine (R-spine), which was discovered already 15 years ago in 2006 [6]. The R-spine consists of four residues, named RS1–RS4, which connects the two lobes of the kinase domain (Figure 1A,B). Two of these residues, RS1 and RS2, belong the C-lobe. RS1 is His (sometimes Tyr) residue from the HRD (or YRD) motif [7]. RS2 is Phe (or Leu) from the DFG-motif, which is part of the activation loop of a protein kinase. The other two R-spine residues belong to the N-lobe of the protein kinase. RS4 is a residue from the β4-strand, which is less conserved but frequently Leu can be found in this position. Finally, RS3 is located four residues C-terminal from the αC-helix Glu that forms a salt-bridge to the Lys of β3-sheet. RS3 is usually (not always) accessible from the ATP binding site as its side chain lines the active site cleft. Overall, αC-helix, where RS3 is located, has a central role in the kinase activation process [8]. In the catalytically active form of a protein kinase, R-spine is assembled and in the inactive state it is disassembled (Figure 1C). In the active state the location of RS2 as part of the assembled R-spine results in an open and extended conformation of the activation loop (A-loop), while a closed A-loop configuration is preferred in the inactive state. Notably, additional stabilization of the R-spine, such as via in-frame insertions or RS3 mutations [9,10], may result in increased catalytic activity of the protein kinase.

Next to the R-spine in the N-lobe are located three conserved residues, named as Shell (SH) residues (Figure 1C) [9]. These residues, which are usually hydrophobic, have a role in supporting R-spine and are therefore important for kinase activity. One of these residues, SH2, is found close to RS3. This SH2 residue is more commonly known as the gatekeeper residue, which is named due to its role in controlling access to the hydrophobic pocket [11]. This shell residue participates in regulating R-spine dynamics, and gatekeeper mutations may stabilize the R-spine promoting the kinase activation [12].

In recent years, ever increasing efforts have been conducted by the pharmaceutical industry to target protein kinases [13]. These efforts have resulted in numerous small-molecule kinase inhibitors, totalling now over 70 FDA approved small-molecule inhibitors (Figure 1D). According to the Protein Kinase Inhibitors in Clinical Trials database (PKIDB) [14,15], approximately 300 small-molecule kinase inhibitors are either in clinical trials or already approved. Comprehensive reviews of the kinase inhibitor drug discovery and kinase inhibitor development are available [16–18]. Currently, oncology is dominating indication for the kinase inhibitors, but there is potential also in other therapeutic areas such as autoimmune and inflammatory diseases, and degenerative disorders [19].

Here, we review the characteristics of RS3 and its relationship with the neighbouring gatekeeper (SH2) based on the publicly available structural data. We also have a look at RS3 interactions to small-molecule kinase inhibitors, including the approved drugs. Finally, we end the review with available mutational data of RS3.

## RS3 in the human kinome

**Figure 2.**
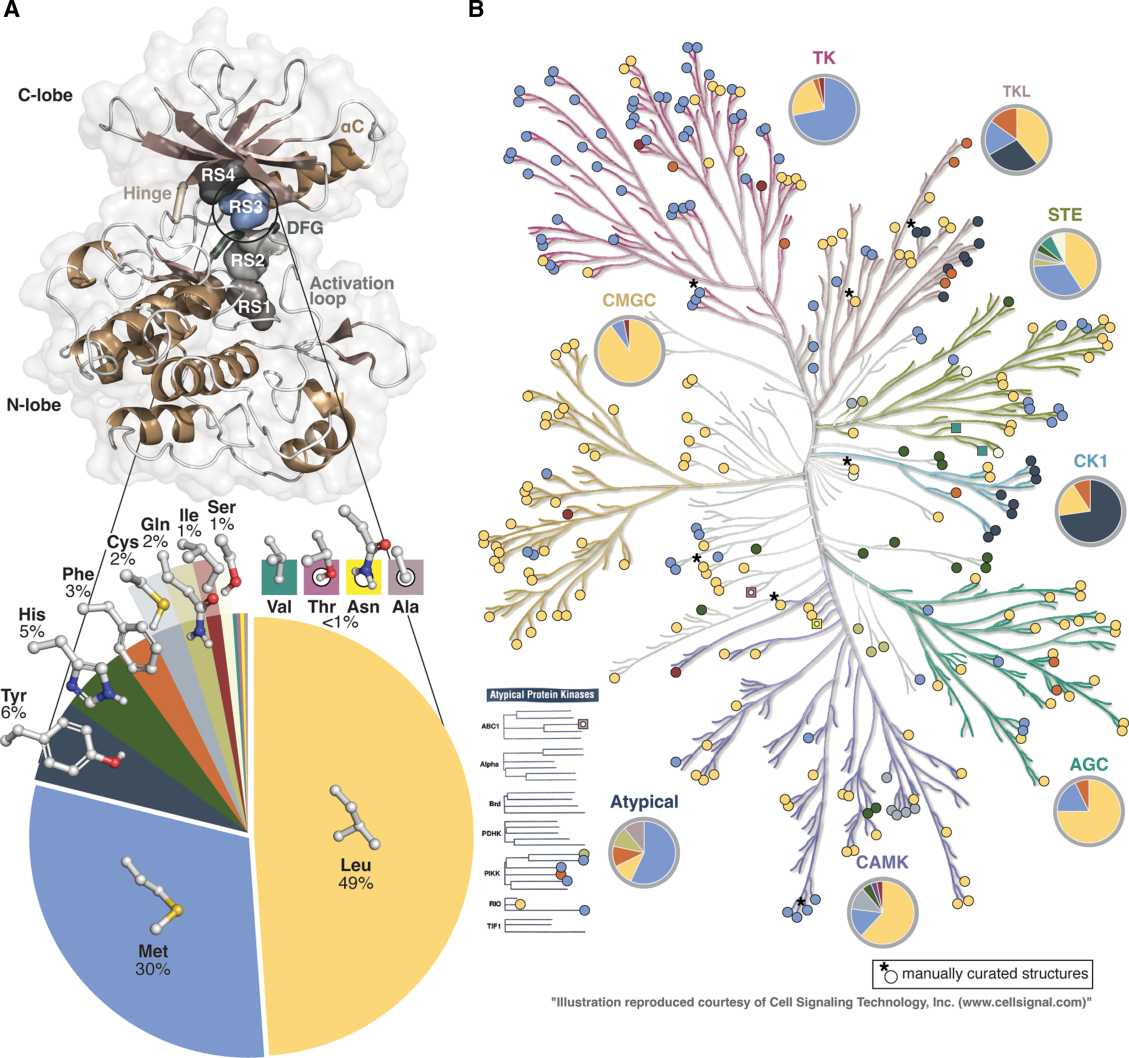
RS3 in human protein kinases with available structures. (**A**) Occurrence of RS3 residues in human protein kinases with publicly available structural data (289 kinases). The location of RS3 is highlighted in IGFR1 kinase domain (PDB ID: 3qqu [28]). The shown frequencies are rounded up to the nearest %, for residues with <1% frequency, percentage is not shown. (**B**) RS3 residue distribution in human kinome. Colours of the residue types are as in **A**. Eight structures with KLIFS annotation errors that were manually curated (RS3 was properly assigned) are indicated with an asterisk. Data in **A** and **B** consist of human protein kinases with publicly available structures with (with lipid kinases excluded). Human kinome tree illustration was made with the help of KinMap [29].

A majority of the human protein kinases with publicly disclosed structures display a nonpolar aliphatic RS3 residue (Figure 2). Nearly half of these kinases exhibit Leu in RS3, and almost a third have Met (Figure 2A). In the overall human proteome Leu is also the most abundant residue (9.97%), while Met has the second lowest frequency (2.13%) of all amino acids [23]. Following the abundant Leu and Met in RS3, next preferred are aromatic residues. Tyr, His and Phe appear with the frequencies of 3–6%. Cys and Gln exist in 2%, Ile and Ser in 1%. Even more rare residues that are observed in this location are Val, Thr, Asn and Ala. The charged residues, Asp, Glu, Lys and Arg, as well as structurally more unique Trp, Gly or Pro are not present in the analysed set of human protein kinases in RS3. Based on the sequence alignment of a larger set of eucaryotic protein kinases these residues have been suggested to exist as RS3 residues, although rarely [7]. Regardless of the high existence of hydrophobic residues in RS3, there exists no clear trend related to the hydrophobicity ranking of the residues and their observed frequencies [24,25].

Protein kinases of different groups and families display distinct preferences for RS3 residues (Figure 2B). The majority of the kinases (72%) belonging to the TK group display Met RS3. More than a fifth (22%) of this group present Leu in this position, including protein kinases belonging to JakA family (JAK1–3; TYK2) and Trk family (NTRK1–3 also known as TRKA–C). Four protein kinases have either Ile (ALK; ERBB3 (ErbB3)) or a Phe (PTK7 (CCK4); LMTK3 (LMR3)). In EGFR family Met is preferred in RS3, except ErbB3 has Ile. Interestingly, ErbB3 has been identified to display considerably lower kinase activity [26,27]. However, ErbB3 displays also other unique characteristics that differ from other EGFRs (for instance, instead of αC-Glu ErbB3 has a His in this location). In the PDGFR family, KIT displays Leu instead of Met that is observed in other family members (FLT, PDGFRA, CSF1R (FMS)).

In contrast with the TKs, in the CMGC group Leu is clearly dominating RS3 (90%). Only three protein kinases have Met in RS3 (MAPK8 (JNK1); MAPK10(JNK3); GSK3B), and MAPK6 (Erk3) displays Ile in this position.

The TKLs prefer quite diversely Leu, Met, Tyr and Phe in their RS3. Members of the STKR family prefer an aromatic residue in this position: Tyr is observed in ACVR1 (ALK2), ACVR2A (ACTR2), ACVRL1 (ALK1), BMBR1B, BMBR2 and TGFBR1 (TGFbR1); Phe in ACVR2B (ACTR2B) and TGFBR2 (TGFbR2). Aromatic Phe is also present in two MLK family members: MAP3K9 (MLK1) and MAP3K21 (MLK4), while other kinases in this family display Leu (ILK; MAP3K12 (DLK); MAP3K20 (ZAK); MAP3K7 (TAK1); TNNI3K (HH498)).

Kinases of the STE group display mainly Leu and Met in RS3. However, also other RS3 residues are observed in this group, including His (STE11) and Ser (STRADA (STLK5); MAP2K6), Gln (MAP3K8 (COT)), Cys (MAP3K14 (NIK)) and Val (MAP2K4; MAP2K7). From these, MAP3K14 (NIK) and MAP3K8 (COT) belong to the STE-Unique family. Three members of the STE7 family display rare RS3 residues. These kinases are MAP2K6 (Ser), MAP2K4 also known as MKK4 (Val) and MAP2K7 also known as MKK7 (Val). Interestingly, unique autoinhibited conformational states have been reported for these kinases state [30–32]; with MKK4 this state may be related to its dimer form [33].

In the CK1 group, Tyr is dominating in RS3 (73%). VRK3 displays an aromatic Phe in this position. TTBK family kinases (TTBK1, TTBK2) are more diverse in this group with their aliphatic Leu in RS3.

The majority of the AGC and CAMK group kinases exhibit Leu in RS3, which is followed by Met with lower frequencies. Two kinases with an aromatic RS3 (Phe) are observed in AGC group, in PKN2 and PRKCI (PKCi). In CAMK group, kinases of the CAMK2 family (CAMK2A, CAMK2B, CAMK2D, CAMK2G) display Cys in RS3, as well as CASK. His is observed in MAPKAP family (MAPKAP2, MAPKAP3). TRIB1 (Trb1) represents Ile in RS3; however, in this pseudokinase the neighbouring Tyr may actually occupy the canonical RS3 position [34,35].

The protein kinases that are not belonging to any specific group display also family specific preferences. For instance, protein kinases of WEE family and PLK family exhibit His in RS3 (located above and below CK1 group in the kinome tree). Of the Atypical kinases, COQ8A that is also known as ADCK3 [36], is the only structure in the dataset that displays Ala in RS3.

## Polar RS3 are rare

Not only hydrophobic RS3 residues exist, but also polar residues are observed in this position. AURKA is an example of a widely studied kinase that has a polar RS3 (Gln) is. It was disclosed by Levinson et al. that this polar residue has a specific role in AURKA activation via a water-network [37]. Similarly, AURKB and AURKB have also Gln as their RS3. In addition, Gln is observed in MAP3K8 (COT, TPL2) and ATM. MAP3K8 controls inflammation [38] and ATR DNA damage responses [39].

In the available data, Ser is observed in three kinases. While in STRADA (STLK5, STE20) this residue appears unreachable from the binding cleft (PDB ID: 3gni [40], 2wtk [41]), in Haspin Ser is accessible (participates in water coordination next to αC-Glu (PDB ID: 4ouc [42]). RS3 Ser may also be accessible in MAP2K6 when it is not in its autoinhibited state (PDB ID: 3fme). In the autoinhibited state its neighbouring Met appears to take the regular RS3 position (PDB ID: 3vn9 [32]).

Two unique polar RS3 residues are present in the data. Asn is observed in the RS3 of CHK1, while all the other kinases belonging to the same CAMKL family have either a lipophilic Leu or Met in this position. CHK1 inhibition could be useful in the treatment of KRAS driven pancreatic ductal adenocarcinoma [43]. Thr is observed in ULK4, while other members of the ULK family (ULK1–ULK3) have Leu in the respective position. ULK4 is a pseudokinase and has an unusual structural characteristic in its αC-helix: it exhibits Trp residue in the location of αC-Glu. This Trp appears to participate in its R-spine formation [44].

## RS3 relationship with gatekeeper

**Figure 3.**
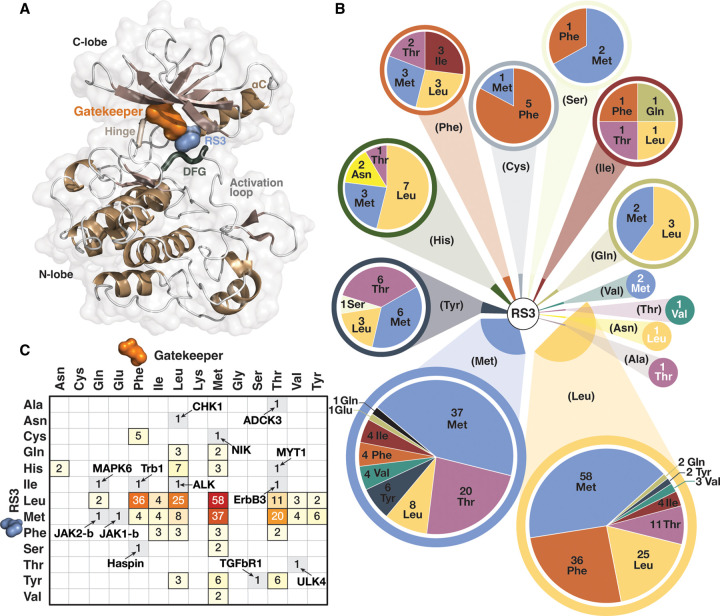
Gatekeeper and RS3. (**A**) Gatekeeper is located near RS3, and in some cases these residues are in contact. In the figure, gatekeeper of the protein kinase IGF1R (PDB ID: 3qqu [28]) is shown with orange surface and RS3 with blue surface. (**B**) Distribution of gatekeeper residues according to their RS3 residue (in parenthesis) represented in pie charts. (**C**) Correlation matrix of RS3 and gatekeeper residues. The unique RS3-gatekeeper combination containing kinases are labelled.

The access towards the hydrophobic pocket (and towards RS3) is controlled by gatekeeper, also known as SH2 residue. This residue may also influence R-spine dynamics and it can be found in close contact to RS3 (Figure 3A). Generally, protein kinases prefer Met, Leu, Phe and Thr gatekeepers (Figure 3B,C). In the available structures, Met is the most abundant gatekeeper (40%), followed by Leu (18%), Phe (16%) and Thr (15%). Less frequent gatekeepers — but presented in more than eight kinase domains — are Ile 4%, Tyr 3%, Val 3%.

Kinases with different RS3 residues display also distinct gatekeeper preferences (Figure 3B,C). With Met in RS3, less Phe gatekeepers are observed in comparison with when RS3 is Leu. Bulky aromatic RS3 residues (Tyr, His, Phe) do not exist in combination with aromatic gatekeepers. The kinase domains with Cys in RS3 appear to prefer Phe gatekeepers. Polar Gln and Asn display either Leu or Met as their gatekeepers. While Thr is rare RS3 residue, as a gatekeeper it is common with Leu, Met and Tyr in RS3. In addition to Thr, other polar gatekeepers do exist. Asn gatekeeper is observed only in combination with His RS3 in WEE1 (Wee1) (PDB ID: 3biz [45]) and WEE2 (Wee1B) (PDB ID: 5vdk [46]). Gln gatekeeper is observed in four kinases, including MAPK1 (Erk2) (PDB ID: 5hnv [47]), MAPK3 (Erk1) (PDB ID: 4qtb [48]), MAPK6 (Erk3) (PDB ID: 7aqb [49]), and pseudokinase domain JAK2-b (PDB ID: 4fvq [50]). Pseudokinase domain Jak1-b has a unique polar Glu as its gatekeeper (PDB ID: 4l00 [51]). Overall, thirteen protein kinases in the analysed dataset display unique RS3-gatekeeper combinations (Figure 3C).

## RS3 and small-molecule kinase inhibitors

**Figure 4.**
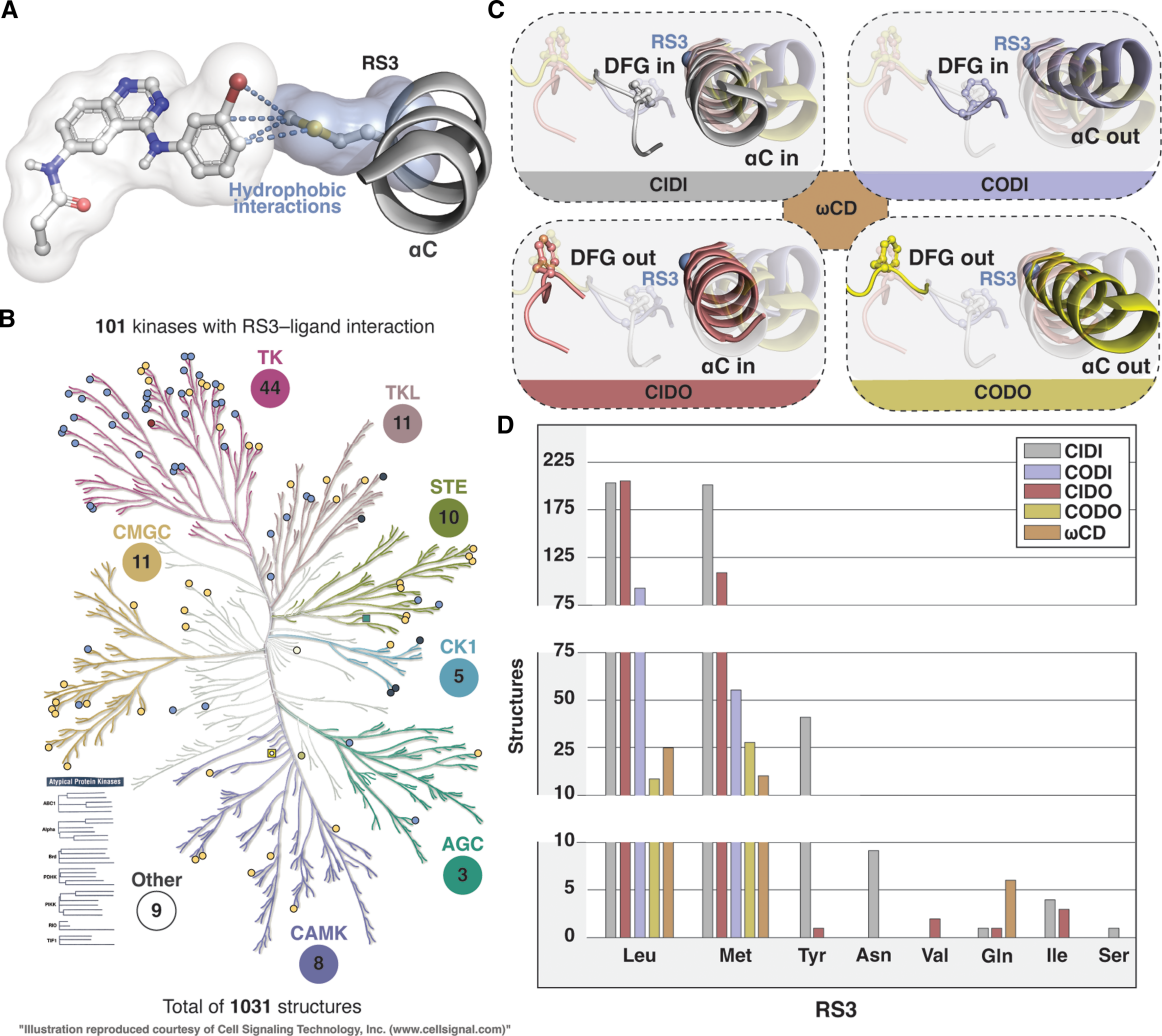
Publicly available structures that display RS3–ligand contacts. (**A**) An example of a structure displaying hydrophobic contacts to RS3 (PDB ID: 4lqm [54]) (**B**) Structures with RS3–ligand interactions. Results of the KLIFS search, which was complemented with Protein–Ligand database (PLDB) (Schrödinger LLC) that identified 197 additional structures. (**C**) Conformation classification of protein kinases based on DFG and αC-helix conformations as disclosed by Ung et al.[55]. The ambiguous conformations are named as ωCD, which may represent the transition conformations between the four states. Location of RS3 Cα-atom is illustrated with a blue sphere. (**D**) Number of structures with RS3–ligand contacts and divided by RS3 residue type and KinaMetrix [56] defined kinase conformations. For 11 structures the conformation was undefined and those were excluded.

We searched the KLIFS database [52] and complemented our search using Protein–Ligand Database (PLDB) tool of Maestro (Schrödinger LLC) to map out all the existing protein kinase–ligand complexes that have contacts between RS3 and the ligand (Figure 4). In KLIFS, RS3 is named as residue #28 (αC-Glu is #24) [53]. Over 100 protein kinases have structures where RS3–ligand interactions are observed (Figure 4B). In total, more than 1000 structures with RS3–ligand interactions are available.

RS3–ligand interactions are observed with kinases containing Leu, Met, Tyr, Asn, Val, Gln, Ile or Ser in their RS3. No direct RS3–ligand contacts were observed for kinases with Ala, Cys, Phe, His and Thr. Polar interactions to RS3 are extremely rare. H-bond interaction occurs between inhibitor and RS3 only in 12 structures, including AURKA (Gln) (PDB ID: 4uzd [57]); CHK1 (Asn) (PDB IDs: 4fsq, 4fst, 4ftk, 4ftl, 4ftm, 4ftn); Haspin (Ser) (PDB ID: 6z5a); VRK1 (Tyr) (PDB IDs: 6btw, 6cfm, 6cnx, 6npn). Hydrophobic interactions are abundant, and hydrophobic contacts to Leu and Met appear in 551 and 410 structures, respectively. This is not surprising, based on their high frequency in RS3 (79%) among the available structures. Interactions to Tyr appear in 41 structures, while other RS3 residues with interactions are represented each with 10 or less structures.

Interactions to RS3 appear independent on the kinase conformation (Figure 4C,D) [55]. Based on the KinaMetrix [56], ‘αC-helix in' conformations are dominating in the structures. CIDI is the most populated with 459 structures and CIDO appears in 323 structures. CODI and CODO structures with αC-helix out configuration exist in 148 and 42 structures, respectively. The ambiguous ωCD occurs in 47 structures. Leu and Met RS3–ligand interaction structures display all configurations, albeit less structures of CODI (16%), CODO (4%) and ωCD (4%) conformations exists. With less structural information containing RS3 residues the conformational representation does not cover all configurations. Nevertheless, all conformations are present in the structures with RS3–ligand interactions. As there exists distinct protein kinase conformation classifications, we also analysed the conformational distribution of RS3 contact structures with Kincore [58]. Table 1 shows the conformational distribution of these structures assigned with the Kincore. Overall, majority of the available compounds with the RS3 interactions exist in DFGin and DFGout spatial classes, covering different conformational classes (dihedrals).

**Table 1:** RS3–ligand contact structures with Kincore defined conformations

Spatial Label	Dihedral label	Annotation	Leu^1^	Met^1^	Tyr	Asn	Val	Gln	Ile	Ser
**DFGin**	BLAminus	Active	71	96	39	9	-	-	-	1
	BLBplus	SRC-inactive	120	50	-	-	-	-	-	-
	ABAminus	Active-like	21	21	1	-	-	-	3	-
	BLBminus		36	33	-	-	-	-	-	-
	BLAplus	FGFR-inactive	23	39	-	-	-	-	-	-
	BLBtrans	CDK-inactive	-	7	-	-	-	-	-	-
	None		38	12	-	-	-	2	1	-
**DFGinter**	BABtrans		-	-	-	-	-	-	-	-
	None	AURKA-inactive	13	6	-	-	-	3	-	-
**DFGout**	BBAminus	Type-2 binding	151	101	1	-	1	1	2	-
	None	DFGout-like	59	32	-	-	1	2	1	-
**None**	None		13	4	-	-	-	2	-	-
**Total**			545	401	41	9	2	10	7	1

1 Conformation definitions were unavailable in Kincore for six (Leu) and nine (Met) individual structures included in Figure 4D.

## Approved small-molecule kinase inhibitors and RS3

**Figure 5.**
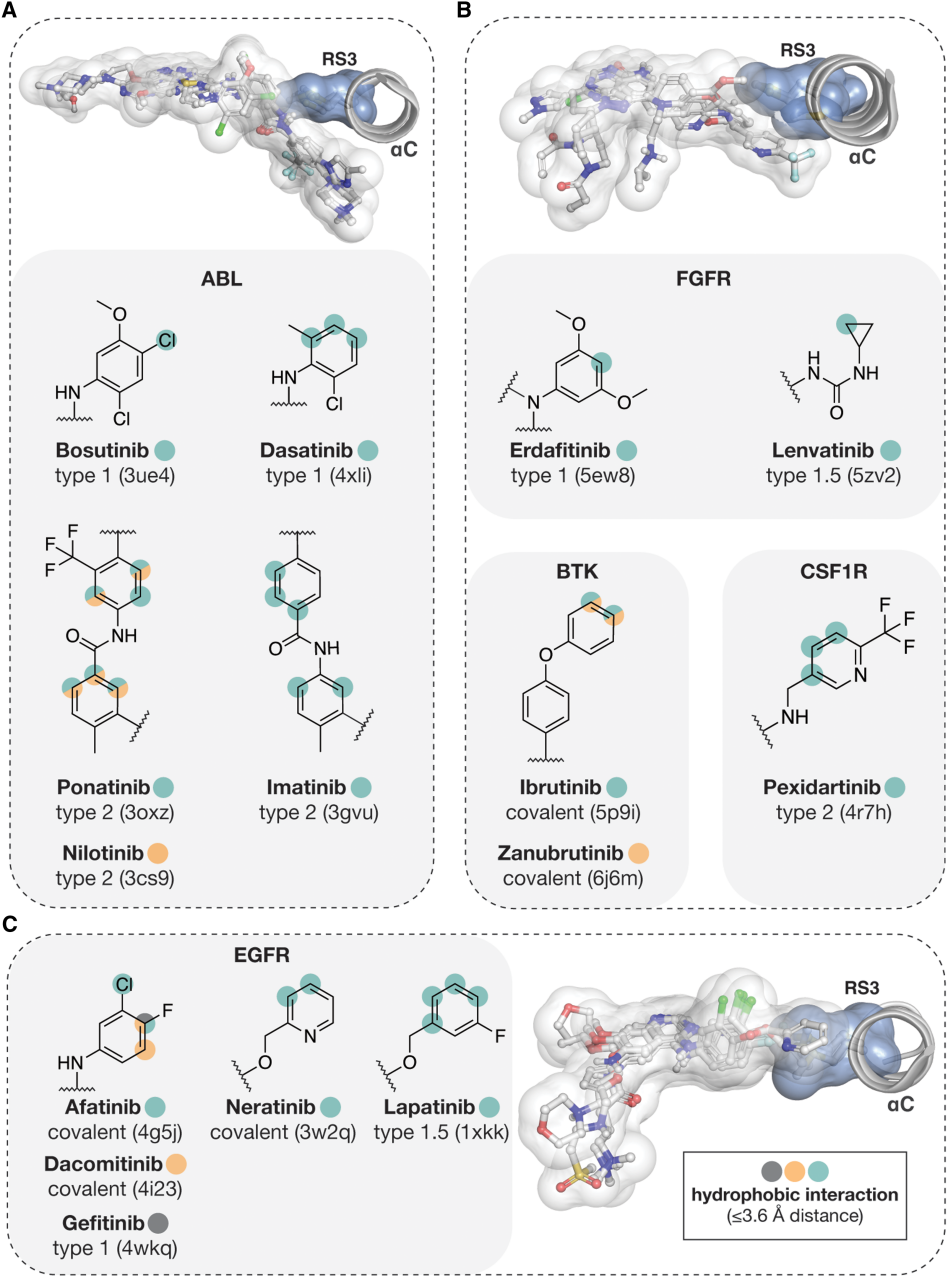
FDA approved small-molecule kinase inhibitors with Met RS3 contacts. (**A**) ABL inhibitors. (**B**) FGFR, BTK and CSF1R inhibitors. (**C**) EGFR inhibitors. In 3D images, structures of the superimposed kinases are shown with their bound inhibitors. Full kinase inhibitor structures are represented with transparent white surface and stick models. RS3 Met is shown with transparent blue surface and sticks. The RS3 interacting atoms of the inhibitors are highlighted in 2D-structures of the structural moieties that are located near the RS3 residue. Hydrophobic interaction was defined based on a 3.6 Å (or shorter) distance between two hydrophobic atoms.

**Figure 6.**
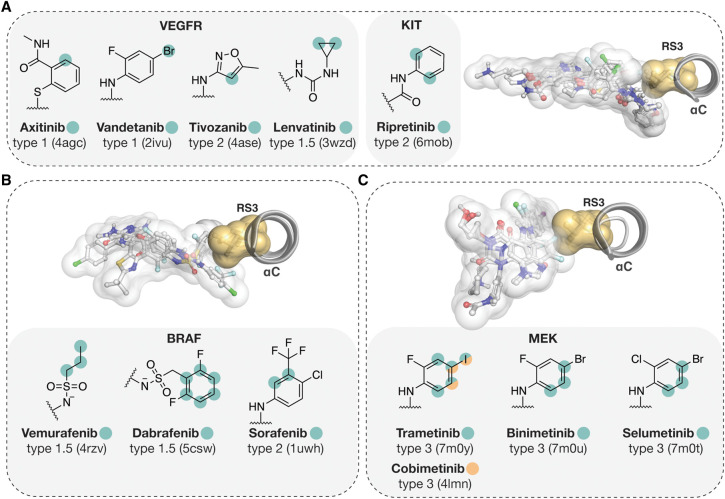
FDA approved small-molecule kinase inhibitors with Leu RS3 contacts. (**A**) VEGFR and KIT inhibitors. (**B**) BRAF inhibitors. (**C**) MEK inhibitors. In 3D images, structures of the superimposed kinases are shown with their bound inhibitors. Full kinase inhibitor structures are represented with transparent white surface and stick models. RS3 Leu is shown with transparent yellow surface and sticks. The RS3 interacting atoms of the inhibitors are highlighted in 2D-structures of the structural moieties that are located near the RS3 residue. Hydrophobic interaction was defined based on a 3.6 Å (or shorter) distance between two hydrophobic atoms.

A publicly available structure exists for 49 out of the 71 FDA-approved small-molecule kinase inhibitors. From these, interaction to RS3 is displayed by 26 inhibitors (55%). These structures include targets with Met (Figure 5) and Leu RS3 residues (Figure 6). Inhibitors which exhibit RS3 interactions represent all types of kinase inhibitors that bind to the ATP-binding cleft. Of note, inhibitors of different type may engage RS3 site in a different manner. For instance, type II inhibitors, which bind the kinase in its inactive conformation, reach beyond the RS3 on the αC-helix side, and thereby can interact with the side chain to its ‘side' or ‘head' or both. Conversely, type I inhibitors, which bind to the active conformation of the kinase, interact mainly with the head of the RS3 side chain.

In occupying the region next to RS3, mainly six membered aromatic ring containing structural moieties are preferred (Figures 5, 6). Correspondingly, these aromatic rings participate in RS3 interactions in most cases. Tivozanib is the only inhibitor that presents a five-membered heteroaromatic ring in this location. Furthermore, aromatic ring attached halogens, Cl with RS3 Met (bosutinib; afatinib) and Br or I with RS3 Leu (vandetanib; trametinib, cobimetinib), display contacts to RS3. Vemurafenib and lenvatinib are the only exceptions that utilize nonaromatic moieties to interact with RS3. In addition to VEGFRs (Leu), lenvatinib also binds and inhibits FGFR-1 (Met). To both, Leu or Met, lenvatinib is in contact from its cyclopropyl urea moiety (PDB IDs: 3wzd [59], 5zv2 [60]). Vemurafenib displays contacts to RS3 Leu from aliphatic carbons of its propylsulfonamide group (PDB ID: 4rzv [61]).

Some of these drugs display interactions with other kinases RS3 that are distinct from their main target. Bosutinib displays hydrophobic contacts to RS3 with MST3 (Leu) (PDB ID: 4qmn [62]), as well as dasatinib to Leu with MAPK14 (p38a) (PDB ID: 3lfa). Ponatinib displays hydrophobic interactions to Leu with RIPK2 (PDB ID: 4c8b [63]), KIT (PDB ID: 4u0i [64]) and BRAF (PDB ID: 6p3d [65]). Nilotinib to Leu with MAPK11 (p38b) (PDB ID: 3gp0). Imatinib displays contact to Leu when bound to KIT (PDB ID: 1t46 [66]) and MAPK14 (p38a) (PDB ID: 3hec [67]). Also, Pexidartinib exhibits contact to RS3 Leu with KIT (PDB ID: 7khg [68]). For inhibitors which main targets display Leu in RS3, apart from lenvatinib no Met RS3 interaction containing structures are available.

RS3 interaction is kinase dependent. Approved drugs with interactions to RS3 do not necessarily exhibit contacts to RS3 with other kinases that they bind to. For instance, gefitinib has been co-crystallized with GAK (Met), where it displays no contact to RS3 (PDB ID: 5y7z [69]). Ibrutinib has been also co-crystallized with MAP2K7 that has Val in RS3, but it does not display any contacts to this residue in this complex (PDB ID: 6yg2 [70]).

## Mutations in RS3 exist rarely

According to the Catalogue of Somatic Mutations In Cancer (COSMIC; v.95) database [71], no clear tendency for mutations in RS3 exists. In total 82 kinases display at least one mutation (missense or silent) (Table 2). Only with ALK, several mutations at this location appear in the data. These mutations include, I1171N, I1171T and I1171S. For BRAF, L505H mutation is found in eight samples. Perhaps the low number of observed RS3 mutations is not surprising, due to the crucial role of this residue in the kinase function. In comparison, RS2 mutations are also rare, with BRAF F595L (13 samples in COSMIC v.95) being the most frequent in the analysed kinases. Meanwhile, RS2 flanking residues are common oncogenic drivers; for instance, BRAF V600E is found in 52 733 samples and EGFR L858R is present in 10 642 samples. Mutations at αC-helix may activate the kinase via destabilizing the kinase inactive conformation [72], but they are mainly found in other locations on the αC-helix than on RS3 [73].

**Table 2:** Missense mutations in RS3 (COSMIC v.95)

	Kinase	RS3	RS3-contact^1^	Mutation																
				N	Q	M	D	T	S	V	I	R	F	K	H	W	P	L	-^2^	Total
	**TK**																			
1	ABL1	M290	+									1								1
2	ALK	I1171	+	18				11	5										2	36
3	DDR2	M629	+							2										2
4	EGFR	M766	+		1			1			1							1		4
5	FGFR2	M538	+								1	1						1		3
6	FLT1	L882	+																1	1
7	JAK2	L902	+														1			1
8	TYK2	L951	+																1	1
9	KDR	L889	+										1						1	2
10	KIT	L644	+																1	1
11	PDGFRa	M648	+								1									1
12	LCK	M292	+								4									4
13	SYK	M424	+					1												1
14	AXL	M589	-					2			1			2						5
15	EphA3	M674	-								2									2
16	EphA7	M686	-					1												1
17	EphB3	M686	-								1									1
18	ErbB3	I744	-					2												2
19	ITK	M410	-								1									1
20	JAK3	L875	-												2		1		5	8
21	ROR2	M526	-							1										1
22	ROS1	M2001	-															1		1
23	ZAP70	M390	-					1												1
	**TKL**																			
				N	Q	M	D	T	S	V	I	R	F	K	H	W	P	L	-	
24	MAP3K7	L81	+										1							1
25	BRAF	L505	+												8					8
26	ACVR2B	F234	-																4	4
27	MAP3K9	F195	-																3	3
28	RAF1	L397	-							1										1
29	HH498	L513	-								2		1				1			4
	**STE**																			
				N	Q	M	D	T	S	V	I	R	F	K	H	W	P	L	-	
30	PAK1	M319	+					1				1								2
31	PAK4	M370	+					1												1
32	STK10	L85	+														1			1
33	SLK	L83	+								1								1	2
34	MAP2K1	L118	+							1									2	3
35	MAP2K6	S103	-										2							2
36	MAP3K14	C444	-									1								1
37	OXSR1	M67	-								1									1
38	PAK5	M498	-								1									1
39	TNIK	L73	-													1				1
	**AGC**																			
				N	Q	M	D	T	S	V	I	R	F	K	H	W	P	L	-	
40	AKT2	L204	+																1	1
41	ROCK1	M128	+								1									1
42	AKT1	L202	-																2	2
43	CDC42BPB	L128	-			1														1
44	DMPK1	L123	-														1		1	2
45	GPRK5	L238	-																1	1
46	MASTL	L85	-																1	1
47	PRKCh	L407	-																1	1
48	SGK1	L150	-						2	1			1						2	6
	**CAMK**																			
				N	Q	M	D	T	S	V	I	R	F	K	H	W	P	L	-	
49	CHEK1	N59	+				1													1
50	DRAK2	L84	+																1	1
51	PIM1	L93	+							1									3	4
52	RPS6KA3	L467	+								1		1							2
53	CAMK1d	L73	-																1	1
54	CAMK4	L93	-								3	1	3							7
55	MARK2	M104	-							1										1
56	MNK1	L98	-							2										2
57	MNK2	L133	-			1														1
58	SgK085	M155	-								2									2
59	PHKg2	L81	-										1		1					2
60	AMPKa2	L68	-																1	1
61	RPS6KA2	L113	-										2							2
62	SNRK	M67	-								1									1
63	STK17B	L84	-																1	1
	**CDK**																			
				N	Q	M	D	T	S	V	I	R	F	K	H	W	P	L	-	
64	GSK3B	M101	+															1		1
65	JNK3	M115	+							1										1
66	Erk5	L106	+																2	2
67	CDK4	L60	-		1												3			4
68	CDK6	L65	-																2	2
69	CK2a1	L85	-							1										1
70	DYRK1A	L207	-							1	1									2
71	HIPK2	L247	-			1														1
72	JNK1	M77	-								1									1
	**Atypical**																			
				N	Q	M	D	T	S	V	I	R	F	K	H	W	P	L	-	
73	ATM	Q2729	-		1										2					3
74	ATR	M2339	-								1									1
75	ADCK3	A415	-				4													4
	**Other**																			
				N		M	D	T	S	V	I	R	F	K	H	W	P	L	-	
76	TTK	L575	+										1			1				2
77	Haspin	S539	-										1							1
78	NEK7	H86	-							1										1
79	TBK1	L59	-										2							2
80	TLK2	H518	-	2																2
81	ULK1	L67	-																1	1
82	WEE2	H263	-																3	3

1 Hydrophobic interaction between ligand and RS3: +at least one structure with RS3–ligand contact available; - no contacts observed in available structures;

2 Silent mutation.

In the literature, only few cases of studies including RS3 mutations have been reported. With BRAF, a secondary mutation in RS3 (L505H) induces resistance to vemurafenib [74]. Also, in another study resistance to dabrafenib or vemurafenib was demonstrated with BRAF L505H mutation [10]. Moreover, alectinib resistance was reported with I1171S and I1171N mutations in ALK [75]. The I1171N mutation was demonstrated to increase autophosphorylation level of ALK *in vitro* [76]. I1171T has been identified to induce crizotinib resistance [77]. Interestingly, these two inhibitors, crizotinib and alectinib do not display direct contacts to RS3. EGFR M766T mutation was reported to induce resistance to gefitinib and erlotinib [78].

## Conclusions

The function of the R-spine and the role of RS3 is quite conserved with typical protein kinases. Couple of residues are dominating RS3 in the available protein kinase domain structures. Nevertheless, also unique RS3 residues are observed, and in combination with gatekeeper even more kinase specific profiles for these residues are observed. Obviously, even with identical RS3–gatekeeper combinations the 3D-environment within this region can be quite different between two kinases. Kinase specific angles and absolute positions of these residues may provide important opportunities for selective targeting. Obviously, one must carefully consider this case-by-case, as RS3 is not targetable in all kinases. With pseudokinases [79–81], which compared with regular protein kinases can vary more in their structure in this region, the role of RS3 and its targeting would require further research.

The general understanding of RS3–ligand interactions are quite limited, even though numerous structures that contain these mainly hydrophobic interactions are available. Currently, no studies investigating specific effect of RS3 on ligand binding affinity exist that directly compare a set of ligands with selected mutations of this residue. Further research is needed to disclose the influence of RS3 residue for ligand binding and should be also extended to the cases where no direct contact between the residue and inhibitor exists. Of note, even with hydrophobic interactions (in the case of hydrophobic RS3) this should not be overlooked as these interactions may be crucial for the inhibitor binding [82]. For example, non-canonical interactions play a detrimental role in binding affinity of the ultra-potent small-molecule biotin [83]. There may be good possibilities available to optimize RS3-specific interactions, for instance, with enhanced interactions with the sulfur atom of Met [84].

Infrequency of mutations in RS3 may indicate a defiance against plausible point mutation in this position that could cause drug resistance [85]. Perhaps the somewhat buried location of RS3 in the quite rigid αC-helix position that offers a limited flexibility, renders the mutations in this location (at least in most cases) incompetent to drive kinase activation. This motivates further to optimize protein–ligand interactions for RS3. However, the data at hand may not necessarily cover potential drug therapy induced mutations in cancer patients. We believe that in near future, with accumulation of this data, this information will be more accessible, and a better estimate can be provided.

The full data presented in this review are freely available at https://doi.org/10.5281/zenodo.5796550

## Perspectives

- The role of the conserved R-spine and RS3 residue in protein kinase function is well established.
- Protein kinases display diversity in their RS3 residue and in its surroundings. Many small-molecule protein kinase inhibitors, including approved drugs, display contacts to RS3.
- Considering the RS3 residue more carefully in the design of small-molecule kinase inhibitors may offer important advantage for the inhibitor binding and selectivity.

## Abbreviations

ATP: adenosine triphosphate
PLDB: Protein–Ligand Database
